# Metabolic effects of skeletal muscle-specific deletion of beta-arrestin-1 and -2 in mice

**DOI:** 10.1371/journal.pgen.1008424

**Published:** 2019-10-17

**Authors:** Jaroslawna Meister, Derek B. J. Bone, Grzegorz Godlewski, Ziyi Liu, Regina J. Lee, Sergey A. Vishnivetskiy, Vsevolod V. Gurevich, Danielle Springer, George Kunos, Jürgen Wess

**Affiliations:** 1 Molecular Signaling Section, Laboratory of Bioorganic Chemistry, National Institute of Diabetes and Digestive and Kidney Diseases, Bethesda, MD, United States of America; 2 Laboratory of Physiologic Studies, National Institute on Alcohol Abuse and Alcoholism, Bethesda, MD, United States of America; 3 Department of Pharmacology, Vanderbilt University, Nashville, TN, United States of America; 4 Murine Phenotyping Core, National Heart, Lung, and Blood Institute, Bethesda, MD, United States of America; Stanford University School of Medicine, UNITED STATES

## Abstract

Type 2 diabetes (T2D) has become a major health problem worldwide. Skeletal muscle (SKM) is the key tissue for whole-body glucose disposal and utilization. New drugs aimed at improving insulin sensitivity of SKM would greatly expand available therapeutic options. β-arrestin-1 and -2 (Barr1 and Barr2, respectively) are two intracellular proteins best known for their ability to mediate the desensitization and internalization of G protein-coupled receptors (GPCRs). Recent studies suggest that Barr1 and Barr2 regulate several important metabolic functions including insulin release and hepatic glucose production. Since SKM expresses many GPCRs, including the metabolically important β_2_-adrenergic receptor, the goal of this study was to examine the potential roles of Barr1 and Barr2 in regulating SKM and whole-body glucose metabolism. Using SKM-specific knockout (KO) mouse lines, we showed that the loss of SKM Barr2, but not of SKM Barr1, resulted in mild improvements in glucose tolerance in diet-induced obese mice. SKM-specific Barr1- and Barr2-KO mice did not show any significant differences in exercise performance. However, lack of SKM Barr2 led to increased glycogen breakdown following a treadmill exercise challenge. Interestingly, mice that lacked both Barr1 and Barr2 in SKM showed no significant metabolic phenotypes. Thus, somewhat surprisingly, our data indicate that SKM β-arrestins play only rather subtle roles (SKM Barr2) in regulating whole-body glucose homeostasis and SKM insulin sensitivity.

## Author summary

Type 2 diabetes (T2D) has become a major health problem worldwide. The key feature of T2D is that blood glucose levels are elevated above the normal range. When blood glucose levels rise, insulin is released from the pancreas, triggering the rapid removal of glucose from the blood. Because of the large mass of skeletal muscle tissue (SKM), insulin-stimulated glucose uptake into SKM is essential for maintaining normal blood glucose levels. In T2D, insulin-stimulated glucose uptake by SKM is significantly impaired. Recent research has shown that two intracellular proteins known as β-arrestin-1 and -2 (Barr1 and Barr2, respectively) play important roles in regulating blood glucose levels. In this study, we investigated the potential roles of SKM Barr1/2 in maintaining normal blood glucose levels. We generated and analyzed several mutant mice that lacked either Barr1 or Barr2 or both Barr1 and Barr2 selectively in SKM. Surprisingly, we found that SKM β-arrestins play only rather subtle roles (SKM Barr2) in regulating blood glucose levels and the responsiveness of SKM to insulin. These findings are important for developing novel anti-diabetic drugs that act by promoting glucose uptake into SKM.

## Introduction

Worldwide, more than 400 million adults are predicted to suffer from type 2 diabetes (T2D), indicating that T2D has become a growing health threat in both industrialized and developing countries [1]. T2D is characterized by increased blood glucose levels due to impairments in insulin release from pancreatic β-cells and insulin resistance, which is defined as decreased action of insulin on insulin-sensitive tissues like skeletal muscle (SKM), adipose tissue, and liver [2]. The major driver behind the current epidemic in T2D is the steady increase in the number of obese individuals, due to changes in lifestyle and diet [3, 4]. Since long-lasting weight loss is difficult to achieve, novel antidiabetic drugs with increased efficacy and reduced side effects are urgently needed.

SKM is the key tissue for whole-body glucose disposal and utilization, being responsible for nearly 70% of insulin-stimulated glucose clearance [5]. Moreover, SKM insulin resistance is considered to be the primary defect in the onset of T2D [2]. Thus, a better understanding of mechanisms that lead to SKM insulin resistance may lead to the development of novel antidiabetic drugs that act by improving SKM insulin sensitivity. Such drugs are not available at present.

Arrestin 2 and 3, also known as β-arrestin-1 and β-arrestin-2 (Barr1 and Barr2, respectively), are two ubiquitously expressed intracellular proteins involved in the regulation of many important physiological functions [6–12]. Barr1 and Barr2 are well known for their role in mediating G protein-coupled receptor (GPCR) desensitization and internalization [13]. One of the best characterized GPCR-β-arrestin interactions is the Barr2-mediated desensitization and internalization of the β_2_-adrenergic receptor (β_2_-AR) [13, 14], a GPCR that plays a key role in regulating SKM glucose fluxes [15, 16]. However, accumulating evidence suggests that β-arrestins can also act as signaling molecules in their own right [17–20].

Recent studies with global Barr1 and Barr2 knockout (KO) mice indicated that β-arrestins play important roles in the regulation of whole-body glucose homeostasis [7, 8, 12]. For example, Barr1 is predicted to have a protective role in the development of diet-induced obesity [8, 21], while Barr2 can ameliorate insulin resistance through an AKT-dependent mechanism [7]. Since the two β-arrestins are widely expressed, it is difficult to delineate the cellular mechanisms underlying the metabolic phenotypes displayed by the global Barr1 and Barr2 KO mice.

By generating and analyzing conditional, cell type-specific Barr1- and Barr2-KO mice, we recently elucidated the roles of the two β-arrestins in several metabolically relevant tissues/cell types including adipocytes [22], hepatocytes [10], and pancreatic β-cells [11, 23]. At present, little is known about the metabolic roles of Barr1 and Barr2 expressed by SKM, despite the central importance of SKM in maintaining whole-body glucose homeostasis. To address this question, we generated SKM-specific Barr1- and Barr2-KO mice and subjected these mutant mice to a series of metabolic tests. Somewhat surprisingly, our data indicate that SKM β-arrestins (Barr2) play only rather subtle roles in regulating whole-body glucose homeostasis.

## Results

### SKM-specific deletion of Barr1 in mice has no effect on whole-body glucose homeostasis

Both Barr1 and Barr2 are ubiquitously expressed in mouse tissues showing high mRNA levels in skeletal muscles (SKM) like quadriceps and gastrocnemius (S1A Fig). Studies with whole-body Barr1-KO mice have shown that Barr1 protects against diet-induced obesity and related metabolic impairments [8]. However, the contribution of SKM to this phenotype remains unknown. To study the potential role of SKM Barr1 in regulating whole-body glucose homeostasis, we generated mice lacking Barr1 selectively in SKM (SKM-Barr1-KO mice). This was accomplished by crossing floxed Barr1 mice (Barr1^fl/fl^) [24] with mice that harbored the HSA-Cre(ER^T2^) transgene [25]. Barr1 deletion in SKM was induced by tamoxifen treatment of HSA-Cre(ER^T2^)-positive Barr1^fl/fl^ adult mice. Tamoxifen-injected Cre-negative Barr1^fl/fl^ littermates served as control mice throughout all experiments. Experiments were initiated one week after the last tamoxifen injection.

The induction of Cre activity resulted in a robust and SKM-specific reduction of Barr1 mRNA (S1B Fig) and protein (S1C Fig). The remaining Barr1 mRNA expression in isolated SKM tissues (S1B Fig) is most likely due to other cell types (e.g. fibroblasts) that are present in SKM. After the ablation of Barr1 protein in SKM had been confirmed (S1C Fig), mice (SKM-Barr1-KO mice) were subjected to several metabolic tests.

**Fig 1:**
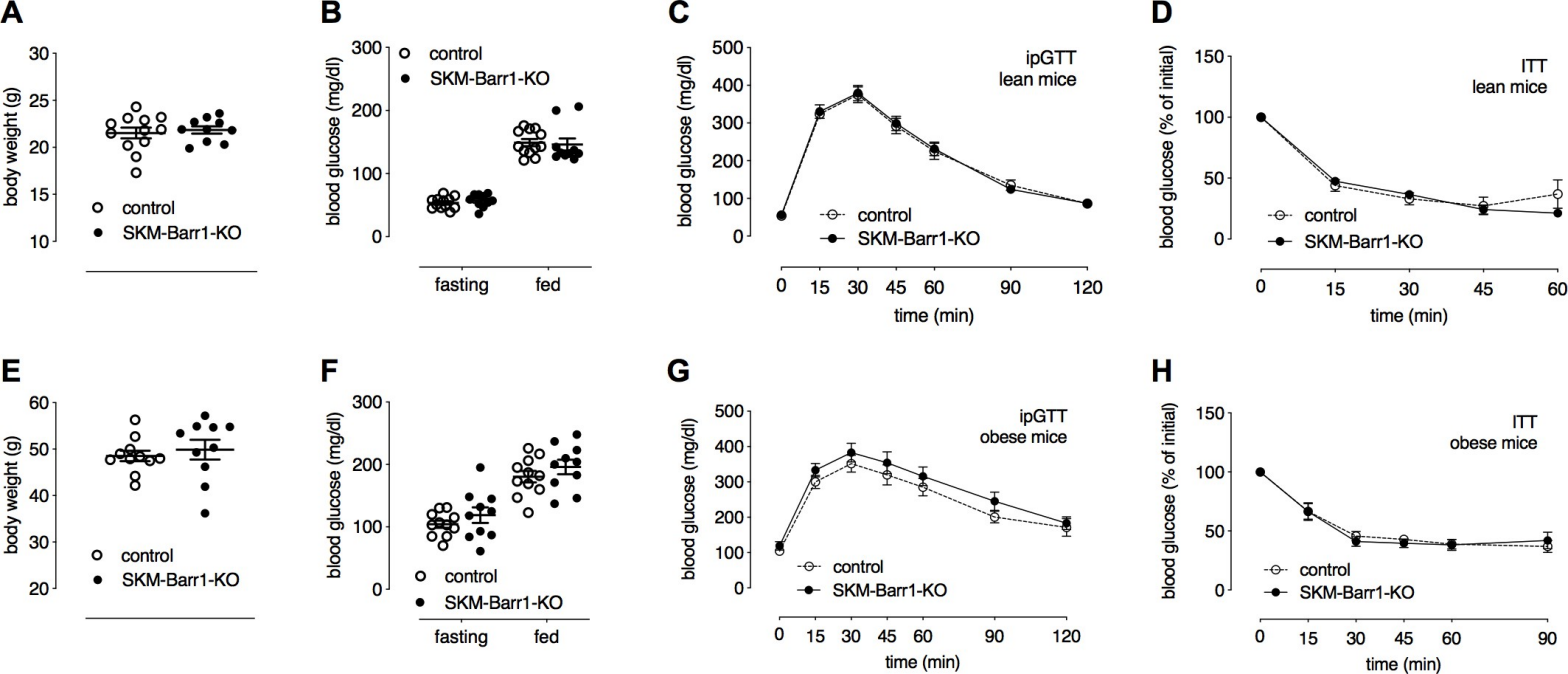
Loss of Barr1 in SKM has no impact on glucose and insulin tolerance in mice. All experiments were carried out with SKM-Barr1-KO mice and their control littermates (adult males) maintained on regular chow (A-D) or a high-fat diet (HFD) for at least 8 weeks (E-H). (A, E) Body weights. (B, F) Fasting and fed blood glucose levels. (C, G) I.p. glucose tolerance test (ipGTT). (D, H) Insulin tolerance test (ITT). Initial blood glucose levels were set to 100% (actual basal blood glucose levels were (in mg/dl): 134 ± 8 vs. 128 ± 6 (D) and 180 ± 9 vs. 196 ± 11 (H) for control vs. SKM-Barr1-KO mice, respectively). Data are shown as mean ± SEM (9–12 mice per group).

Interestingly, SKM-Barr1-KO mice kept on standard chow showed no differences in body weight (Fig 1A), fasting and fed blood glucose levels (Fig 1B), and glucose and insulin tolerance (Fig 1C and 1D), as compared to their control littermates. To metabolically challenge the mice, we maintained them on a high-fat diet (HFD) for a period of 8 weeks. SKM-Barr1-KO mice and control littermates showed similar weight gain when consuming the HFD (Fig 1E). Moreover, the lack of Barr1 in SKM had no significant effect on fed and fasting blood glucose levels (Fig 1F) and glucose and insulin tolerance (Fig 1G and 1H).

### SKM-specific deletion of Barr2 results in minor improvements in glucose tolerance in HFD mice

Barr2 action in hepatocytes [7, 10] and pancreatic β-cells [11] is essential for maintaining proper glucose homoeostasis. At present, it remains unclear whether Barr2 expressed by SKM cells is involved in regulating glucose homoeostasis and insulin sensitivity. To address this issue, we generated mice that lacked Barr2 selectively in SKM (SKM-Barr2-KO mice) by employing the same strategy as described above for the generation of the SKM-Barr1-KO animals. Barr2 mRNA was selectively reduced in SKM tissues (S1D Fig). The absence of Barr2 protein in SKM was also confirmed by western blotting analysis (S1E Fig).

**Fig 2:**
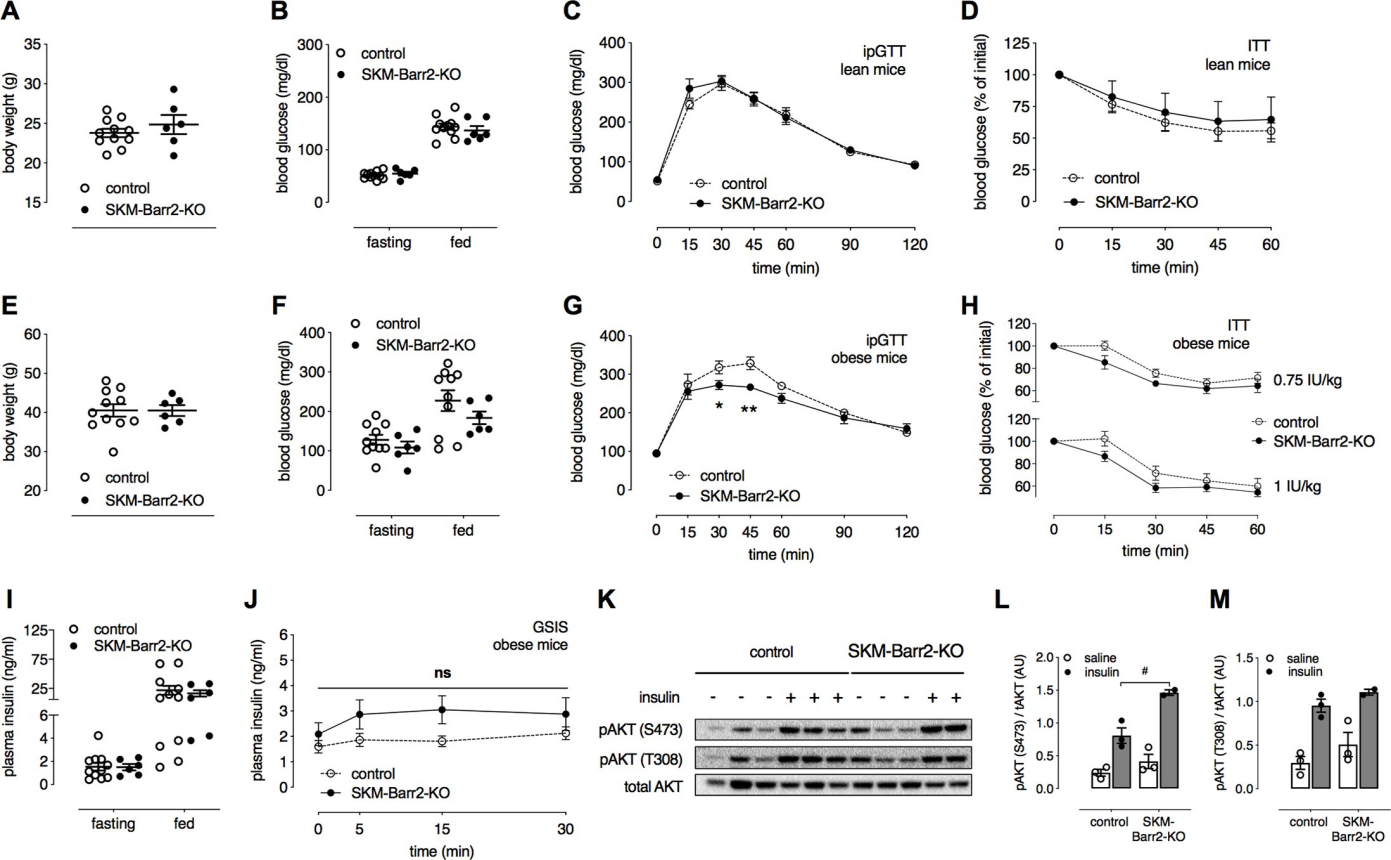
HFD SKM-Barr2-KO mice show improved glucose tolerance. All experiments were carried out with SKM-Barr2-KO mice and their control littermates (adult males) maintained on regular chow (A-D) or a high-fat diet (HFD) for at least 8 weeks (E-M). (A, E) Body weights. (B, F) Fasting and fed blood glucose levels. (C, G) I.p. glucose tolerance test (ipGTT). (D, H) Insulin tolerance test (ITT). Initial blood glucose levels were set to 100% (actual basal blood glucose levels were (in mg/dl): 144 ± 6 vs. 137 ± 9 (D) and 165 ± 7 vs. 177 ± 7 (H, upper panel) and 183 ± 30 vs. 222 ± 16 (H, lower panel) for control vs. SKM-Barr2-KO mice, respectively). (I) Fasting and fed plasma insulin levels. (J) Glucose-stimulated insulin secretion (GSIS). Data are shown as mean ± SEM (6–11 mice per group). *P < 0.05, **P < 0.01 (Student’s unpaired t-test). (K-M) Western blotting studies examining AKT phosphorylation. HFD mice were fasted for 2 h and injected with saline (-) or insulin (0.75 IU/kg). Quadriceps muscles were isolated 10 min later, and protein lysates were used for western blotting studies. (L, M) Quantification of the Western blotting data shown in (K). Data are presented as mean ± SEM. #P < 0.01 (2-way-ANOVA). Ns, no statistically significant difference (2-way-ANOVA).

Like the SKM-Barr1-KO mice, chow-fed SKM-Barr1-KO mice showed unchanged body weight (Fig 2A), fasting and fed blood glucose levels (Fig 2B), and glucose and insulin tolerance (Fig 2C and 2D), as compared to their control littermates. To induce obesity and obesity-associated metabolic deficits such as impaired glucose tolerance and reduced insulin sensitivity, SKM-Barr2-KO mice and their control littermates were maintained on a HFD for at least 8 weeks. Although the two groups of mice showed similar body weights (Fig 2E), fasting and fed blood glucose (Fig 2F) and plasma insulin levels (Fig 2I), SKM-Barr2-KO mice displayed significantly improved glucose tolerance (Fig 2G) and a trend towards enhanced insulin sensitivity (Fig 2H) and glucose-stimulated insulin secretion (GSIS; Fig 2J).

To examine whether Barr2 affected insulin signaling in SKM, we injected obese SKM-Barr2-KO and control mice with either saline or insulin (0.75 IU/kg i.p.) and isolated quadriceps muscles for western blotting studies. Interestingly, the lack of Barr2 in SKM enhanced insulin-induced AKT phosphorylation at S473 (Fig 2K–2M). This result is consistent with enhanced insulin signaling in SKM and the observed improvements in glucose and insulin tolerance in the obese SKM-Barr2-KO mice (Fig 2G and 2H).

**Fig 3:**
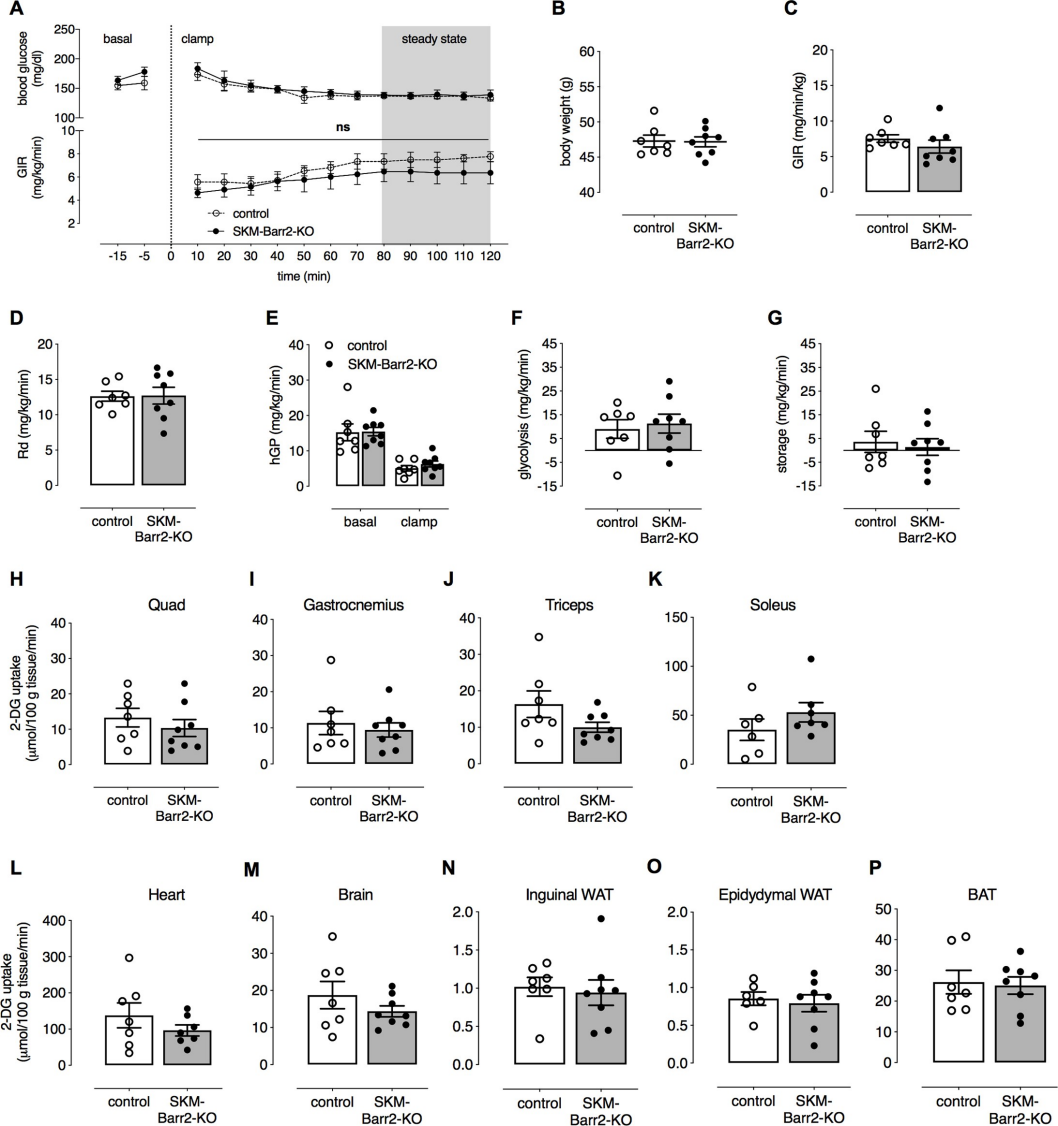
HFD SKM-Barr2-KO mice show no metabolic phenotype in hyperinsulinemic-euglycemic clamp studies. Hyperinsulinemic-euglycemic clamps were performed in conscious, unrestrained HFD control and SKM-Barr2-KO mice (adult males) that had been fasted for 5 h. (A) Time course of blood glucose levels and glucose infusion rates (GIR) during the course of clamps. (B) Body weights. (C) GIR during the steady-state period of the clamp. (D) Whole body glucose clearance (Rd). (E) Hepatic glucose production (hGP) under basal and steady-state conditions. (F) Glycolysis rate. (G) Glycogen storage rate. (H-P) Glucose uptake into SKM tissues (H-K) and other metabolically important tissues (L-P). Quad, quadriceps muscle; WAT, white adipose tissue; BAT, brown adipose tissue. 2-DG tissue uptake was determined as described in Materials and Methods. Data are shown as mean ± SEM (7–8 mice per group). Ns, no statistically significant difference (2-way-ANOVA).

Since metabolic improvements displayed by the SKM-Barr2-KO mice were rather mild (Fig 2G and 2H), we carried out a hyperinsulinemic-euglycemic clamp study as a more robust method to assess insulin sensitivity. Surprisingly, clamp studies with obese SKM-Barr2-KO mice and their control littermates showed that blood glucose levels and glucose infusion rates (GIR; Fig 3A and 3C), whole-body glucose clearance (Rd; Fig 3D), and insulin-mediated suppression of hepatic glucose production (hGP; Fig 3E) during the steady state of the insulin clamp were not affected by SKM-Barr2 deficiency. Similarly, we did not observe any changes in glycolysis and glycogen storage rates between the two groups of mice (Fig 3F and 3G, respectively). To measure *in vivo* glucose uptake into different tissues, we injected obese SKM-Barr2-KO mice and their control littermates with a 10 μCi bolus of [^14^C]2-DG at the end of the clamp. Twenty-five min later, tissues were collected and analyzed. However, no differences in glucose uptake could be detected in SKM and other tissues between the two groups of mice (Fig 3H–3P).

### SKM-specific deletion of Barr2 enhances exercise-mediated glycogen breakdown

β-arrestins are recruited by activated GPCRs, leading to GPCR desensitization and internalization [26]. In SKM, the β_2_-adrenergic receptor (β_2_-AR) is expressed at particularly high levels [27]. Exercise is known to activate the sympathetic nervous system, leading to norepinephrine (epinephrine)-mediated activation of SKM β_2_-AR and the rapid catabolism of energy reserves like glycogen [16, 28].

**Fig 4:**
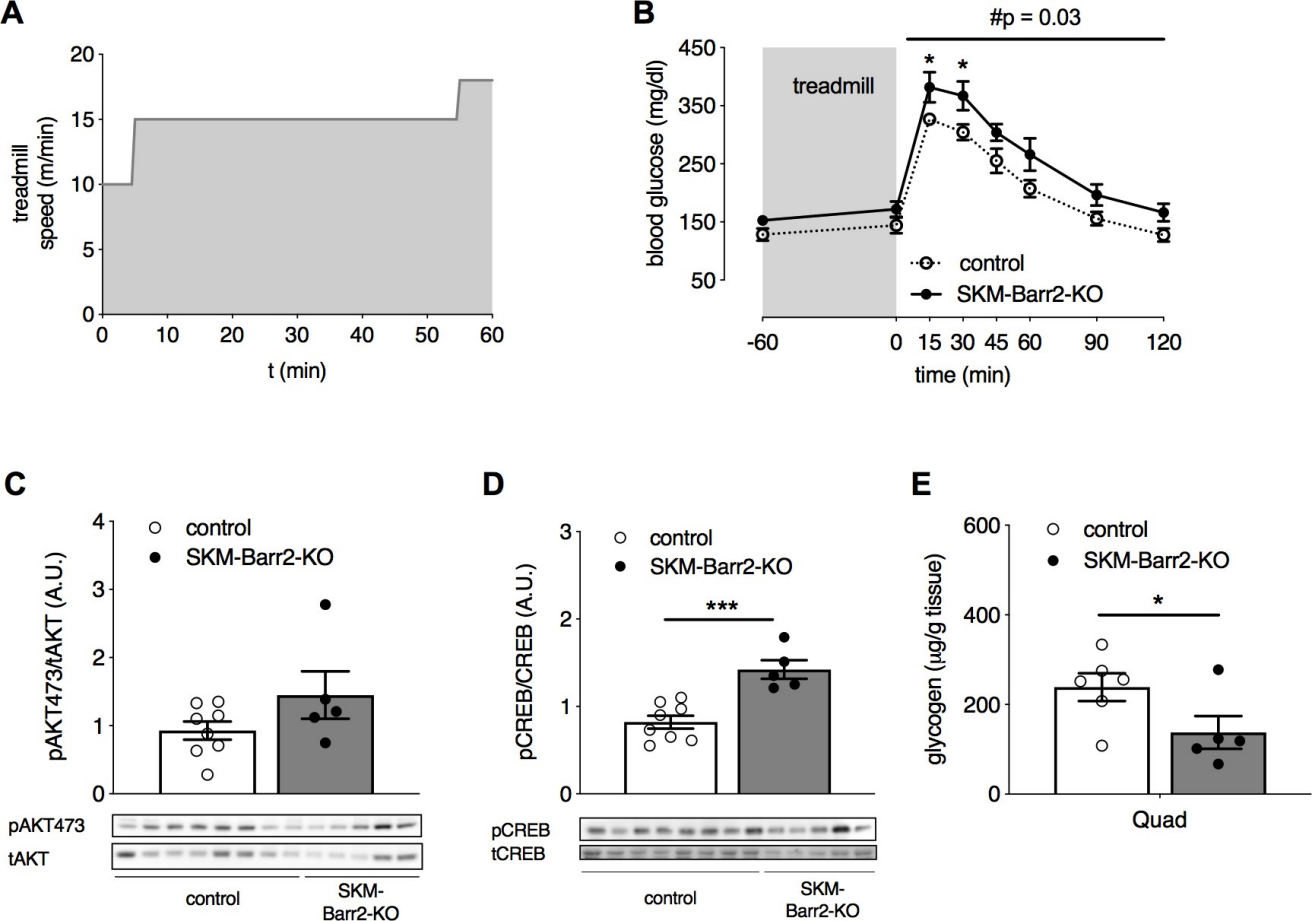
SKM-Barr2-KO mice show increased exercise-induced glycogen breakdown and impaired post-exercise glucose tolerance. Male adult SKM-Barr2-KO and control mice consuming normal chow were run on a treadmill for 1 h following the protocol illustrated in (A). (B) GTT (2 g/kg glucose i.p.) performed after the treadmill exercise. (C-E) Western blotting studies and SKM glycogen content. Mice were subjected to the experimental protocol shown in (A), injected with glucose (2 g/kg glucose i.p.), and then euthanized for the isolation of SKM tissues. (C, D) Western blotting studies. (E) SKM glycogen content. Data are presented as mean ± SEM (5–8 mice per group). *P < 0.05, ***P < 0.001 (Student’s unpaired t-test); #P = 0.03 (2-way-ANOVA). Quad, quadriceps muscle.

To study whether SKM β-arrestins regulate the function of SKM β_2_-ARs, we challenged the SKM-specific β-arrestin KO mice consuming regular chow with a treadmill exercise. SKM-Barr1-KO, SKM-Barr2-KO and their respective control mice that had been fasted overnight were run on a treadmill with increasing speed until the point of exhaustion. Both groups of KO mice displayed similar treadmill performance as their control littermates, showing no significant differences in total running distance, running time, maximum speed reached, completed work, and pre- and post-exercise blood glucose levels (S2 Fig and S3 Fig). To examine whether SKM β-arrestin deficiency altered glucose tolerance following an exercise challenge, mice that had been fasted for 4 h were run on a treadmill for 1 h using the protocol shown in Fig 4A. Blood glucose levels were determined following i.p. injection of a glucose bolus (2 g/kg). While glucose tolerance was not altered in SKM-Barr1-KO mice (S2G Fig), SKM-Barr2-KO mice showed significantly impaired glucose tolerance under these experimental conditions (Fig 4B).

Western blotting studies showed that AKT phosphorylation was not affected by the lack of Barr2 in SKM after exercise testing (quadriceps muscle; Fig 4C). In contrast, the phosphorylation of CREB (cAMP response element-binding protein) was significantly enhanced in SKM lacking Barr2, indicative of increased signaling through the cAMP signaling cascade (Fig 4D).

Since glycogen is the main fuel source during short-term exercise [29] and activation of β_2_-ARs promotes glycogenolysis [15], we measured glycogen levels in mice that were run on a treadmill for 1 h and received a glucose bolus afterwards. We found that glycogen levels were significantly reduced in SKM (quadriceps muscle) of SKM-Barr2-KO, as compared to SKM from control mice (Fig 4E). Enhanced glycogenolysis results in high intracellular levels of glucose-6-phosphate, leading to the inhibition of hexokinase and impaired glucose uptake into SKM due to a decreased glucose gradient across the membrane [15, 30]. Thus, the observation that glycogen breakdown was enhanced in SKM lacking Barr2 (Fig 4E) may explain why SKM-Barr2-KO displayed impaired glucose tolerance after the treadmill exercise (Fig 4B).

### Improvement in glucose tolerance following chronic β_2_-AR stimulation is β-arrestin independent

**Fig 5:**
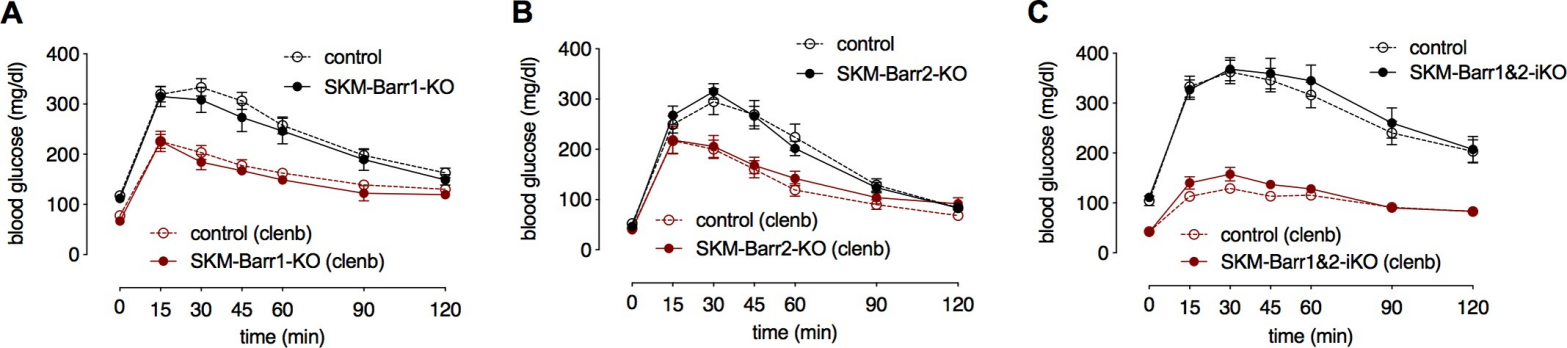
SKM β-arrestins are not required for clenbuterol-mediated improvements in glucose tolerance. Glucose tolerance tests were carried out with male adult mutant mice and their control littermates. Mice were treated with the selective β_2_-AR agonist clenbuterol (clenb) by adding clenb (30 mg/l) to the drinking water for 5 days (red lines). Mice consuming regular drinking water are represented by the black lines. (A) SKM-Barr1-KO and control mice. (B) SKM-Barr2-KO mice and control mice. (C) SKM-Barr1&2 double KO and control mice. Data are shown as mean ± SEM (8–12 mice per group). iKO, inducible KO.

Chronic administration of β_2_-AR agonists, such as clenbuterol, results in beneficial effects on whole-body glucose homeostasis [31]. It has been proposed that these effects may be mediated by β_2_-ARs expressed by SKM [31–35]. To examine the potential role of SKM β-arrestins in modulating the beneficial metabolic effects of β_2_-AR agonists, we treated SKM-Barr1-KO and SKM-Barr2-KO mice and their respective control littermates with clenbuterol drinking water (30 mg/l) for 5 days. Strikingly, clenbuterol-treated control mice showed a dramatic improvement in glucose tolerance (Fig 5A–5C). Interestingly, knockdown of Barr1 or Barr2 in SKM had no significant effect on this metabolic phenotype (Fig 5A and 5B), indicating that β-arrestins do not play a major role in modulating the beneficial metabolic effects of chronic β_2_-AR stimulation (note that residual Barr1/2 expression may still be present).

Since β-arrestins show a high degree of sequence and structural similarity, both isoforms significantly overlap in their function [14]. We therefore wondered whether functional redundancy of the two β-arrestin isoforms might account for the rather mild phenotypes observed with the SKM-specific β-arrestin single KO mice. Hence, we generated mice that lacked both Barr1 and Barr2 selectively in SKM throughout development (SKM-Barr1&2-cKO mice). We also generated mice in which we inactivated both β-arrestins in a tamoxifen-inducible fashion in adult mice (SKM-Barr1&2-iKO mice). Systematic phenotyping studies showed that neither lean nor obese SKM-Barr1&2-iKO and -cKO mice showed any significant metabolic changes, as compared to their corresponding control littermates (Fig 5C, S4 Fig and S5 Fig, respectively). Taken together, these data suggest that SKM β-arrestins do not play a major role in whole-body glucose homeostasis and insulin sensitivity.

## Discussion

Recent studies have shown that β-arrestins play important roles in maintaining glucose and energy homeostasis by regulating the activity of several metabolically important tissues or cell types, including fat, liver, or β-cells [7, 8, 10–12, 21–23]. In the current study, we focused on the potential metabolic roles of Barr1 and Barr2 expressed by SKM. Studies with SKM-specific Barr1-KO mice showed that lack of Barr1 in SKM had no significant impact on glucose tolerance and insulin sensitivity in lean and diet-induced obese mice (Fig 1). These results indicate that the metabolic impairments displayed by global Barr1-KO mice [8] do not involve SKM Barr1 but are most likely the result of increased adiposity caused by the absence of Barr1 in other tissues or cell types.

Interestingly, a recent study showed that chronic clenbuterol treatment stimulated hypertrophic and contractile responses in SKM of WT mice, but not in mice lacking Barr1 [24]. This finding suggests a novel role for Barr1 in mediating β_2_-AR-stimulated SKM growth and strength. On the other hand, we found that SKM Barr1 deficiency did not result in an increase of treadmill exercise capacity in untreated mice (S2A–S2D Fig). However, our *in vivo* data are in agreement with the observation that muscle mass and *ex vivo* muscle force did not differ between WT and global Barr1-KO mice in mice that had not been treated with clenbuterol [24].

Consistent with a previous report [31], we found that treatment of WT mice with clenbuterol for 5 days greatly improved glucose tolerance (Fig 5). This clenbuterol effect did not require the presence of Barr1 in SKM (Fig 5A). This observation clearly indicates that the Barr1-dependent increase in SKM hypertrophy observed after clenbuterol treatment of WT mice for 2–4 weeks [24] involves a mechanism that is distinct from that causing clenbuterol-mediated improvements in glucose tolerance. In agreement with this notion, we found that treatment of WT mice with clenbuterol for 5 days did not cause any changes in SKM mass (lean body mass; Dr. Oksana Gavrilova, personal communication). Like SKM-Barr1-KO mice, SKM-Barr2-KO mice and SKM-Barr1&2 double KO mice showed clenbuterol-induced improvements in glucose tolerance that were similar in magnitude to those displayed by their control littermates (Fig 5). These data clearly indicate that SKM β-arrestins do not modulate the beneficial metabolic effects of β_2_-AR agonists on glucose homeostasis in mice. This finding is surprising since β-arrestins are known to play essential roles in β_2_-AR desensitization and internalization [13].

In contrast to SKM-Barr1-KO mice, SKM-Barr2-KO mice consuming a HFD showed mild improvements in glucose and insulin tolerance (Fig 2G and 2H). However, in hyperinsulinemic-euglycemic clamp studies, HFD SKM-Barr2-KO mice and their control HFD littermates showed similar insulin sensitivity and glucose fluxes (Fig 3). Although the hyperinsulinemic-euglycemic clamp is considered the gold standard in assessing insulin sensitivity [36], this surgically invasive procedure introduces additional stressors and uses non-physiological supramaximal insulin concentrations that might mask small effects in tissue responses to insulin. Moreover, while the clamp work was carried out with mice that had been fasted for only 5 h, the improved glucose tolerance displayed by the HFD SKM-Barr2-KO mice was observed after an overnight (15 h) fast. Prolonged fasting periods increase insulin sensitivity in mice [37], providing a possible explanation for the observation that HFD SKM-Barr2-KO mice did not display any metabolic phenotype in the clamp studies.

While chronic β_2_-AR stimulation with clenbuterol did not require SKM Barr2 to improve glucose tolerance (Fig 5B), the exercise-induced activity of endogenous SKM β_2_-ARs appeared increased in SKM-Barr2-KO mice (Fig 4D), as indicated by increased phosphorylation of CREB, a downstream target of β_2_-AR stimulation [24, 28]. Moreover, SKM-Barr2-KO mice displayed decreased glycogen levels following the treadmill exercise (Fig 4E). Taken together, these data support the concept that lack of Barr2 in SKM increases endogenous β_2_-AR signaling and promotes glycogenolysis. However, under our experimental conditions, these changes in β_2_-AR activity had no significant effect on the performance of the SKM-Barr2-KO mice on the treadmill (S3A–S3D Fig). Nevertheless, our data do not exclude the possibility that the lack of SKM Barr2 may affect exercise capacity under different experimental conditions.

We found that the lack of Barr2 in SKM increased insulin-mediated phosphorylation of AKT in SKM of obese mice (Fig 2K and 2L). In contrast, a study carried out with global Barr2-KO mice reported a decrease in insulin-stimulated AKT phosphorylation in liver, SKM, and adipose tissue [7]. This discrepancy is most likely a consequence of the different mouse models used, and potentially, differences in the genetic background of the mice that were analyzed. In contrast to studies with global Barr2-KO mice, the use of SKM-Barr2-KO mice makes it possible to assess the effect of Barr2 on SKM insulin signaling in an unambiguous fashion. Thus, the impairments in SKM insulin signaling observed with global Barr2-KO mice [7] are most likely caused by the lack of Barr2 in other metabolically relevant tissues that affect SKM insulin signaling in an indirect fashion. Similarly, we recently demonstrated that the liver-specific loss of Barr2 did not affect insulin signaling in the liver [10], in contrast to data obtained with global SKM-Barr2 KO mice [7].

In conclusion, while β-arrestins play key metabolic roles in regulating the function of various metabolically important cell types, including adipocytes [22], hepatocytes [10] and pancreatic β-cells [11, 23], the lack of Barr1 or Barr2 or the combined lack of Barr1 and Barr2 in mouse SKM causes no significant (Barr1) or only relatively minor metabolic phenotypes (Barr2). This is a surprising observation, given the key role of β-arrestins in regulating GPCR function and initiating G protein-independent signaling.

SKM Barr1&2 double KO mice (S4 and S5 Figs) failed to display the moderate metabolic improvements exhibited by SKM Barr2 single KO mice (Fig 2G and 2H). Previous studies have shown that Barr1 and Barr2 can have opposing functional roles in the same tissue or cell type [14], providing a possible explanation for this surprising observation.

Interestingly, the lack of Barr2 in SKM promoted insulin-induced AKT phosphorylation (Fig 2K and 2L). It is likely that enhanced SKM AKT activity is responsible for the improvement in glucose tolerance displayed by the obese SKM-Barr2 KO mice. SKM AKT activity is predicted to play an important role in numerous SKM functions under both physiological and pathophysiological conditions, including SKM atrophy, aging, and cancer [38]. Thus, it should be of particular interest to explore the potential roles of SKM Barr2 in SKM cell differentiation, growth, proliferation, and apoptosis in future studies.

## Materials and methods

### Ethics statement

All animal studies were carried out according to the US National Institutes of Health Guidelines for Animal Research and were approved by the NIDDK Institutional Animal Care and Use Committee.

### Materials

All common chemicals were obtained from Sigma-Aldrich, unless stated otherwise.

### Mouse maintenance and diet

All mice were fed ad libitum and kept on a 12-h light, 12-h dark cycle. Mice were maintained either on a standard mouse chow (7022 NIH-07 diet, 15% kcal fat, energy density 3.1 kcal/g, Envigo Inc.) or a high-fat diet (HFD, 35.5% (w/w) fat content; # F3282, Bioserv) for at least 8 weeks.

### Generation of SKM-specific Barr1- and/or Barr2-KO mice

To selectively inactivate Barr1 and Barr2 in SKM, floxed Barr1 [24] and floxed Barr2 mice [39], in which exon 2 was flanked by two loxP sites, were used (Barr1^fl/fl^ and Barr2^fl/fl^ mice, respectively). These mice were crossed with HSA-Cre(ER^T2^) mice [25] to obtain the HSA-Cre(ER^T2^) Barr1^fl/fl^ and HSA-Cre(ER^T2^) Barr2^fl/fl^ mice used for experimentation. To generate tamoxifen-inducible SKM-specific Barr1 and Barr2 double-knockout (HSA-Cre(ER^T2^) Barr1^fl/fl^&Barr2^fl/fl^) mice, HSA-Cre(ER^T2^) Barr2^fl/fl^ mice were crossbred with Barr1^fl/fl^ mice.

To exclude developmental changes, Cre recombinase activity was induced in 7–8 week-old HSA-Cre(ER^T2^)-positive Barr1^fl/fl^, Barr2^fl/fl^, Barr1^fl/fl^&Barr2^fl/fl^ mice and their Cre-negative littermates (control mice) by intraperitoneal (i.p.) injection with tamoxifen (2 mg per day dissolved in corn oil) for 5 consecutive days.

To generate constitutive SKM-specific Barr1 and Barr2 double-knockout mice (SKM-Barr1&2 cKO mice), Barr1^fl/fl^&Barr2^fl/fl^ mice were crossbred with mice that express HSA-Cre recombinase constitutively (The Jackson Laboratory, B6.Cg-Tg(ACTA1-cre)79Jme/J). Cre-positive Barr1^fl/fl^&Barr2^fl/fl^ and their Cre-negative Barr1^fl/fl^&Barr2^fl/fl^ littermates (control mice) were used for metabolic studies.

All mice used in the present study were backcrossed to the C57BL/6 strain background for at least 5 generations.

### *In vivo* metabolic studies

All metabolic tests were performed with male littermates (age range: 10–30 weeks) using standard protocols. For i.p. glucose tolerance tests (ipGTT), mice were fasted overnight for 15 h, and blood glucose levels were measured before (0 min) and at defined time points after i.p. injection of normal saline containing glucose (2 g/kg for mice consuming normal chow; 1 g/kg for mice on HFD). For insulin tolerance tests (ITT), mice were fasted for 4 h, and blood glucose levels were measured before (0 min) and at indicated time points after i.p. injection of human insulin (0.75 IU/kg or 1 IU/kg as indicated; Humulin R, Eli Lilly). Blood glucose levels were determined using an automated blood glucose meter (Glucometer Elite Sensor; Bayer). To study glucose-stimulated insulin secretion (GSIS), mice were fasted overnight for 15 h, and blood samples were collected in heparinized capillary tubes (Fisher Scientific) before (0 min) and 5, 15, and 30 min following the i.p. injection of glucose (2 g/kg or 1 g/kg as indicated). Samples were centrifuged (5,000 g, 10 min, 4°C) for plasma collection, and plasma insulin levels were determined using an ultra-sensitive mouse insulin ELISA kit (Crystal Chem, Inc).

### Chronic clenbuterol administration

Glucose tolerance tests (ipGTTs) were performed with control and the indicated KO mice as described above. Two days later, mice started to consume drinking water containing clenbuterol (30 mg/l). After 5 days on clenbuterol water, mice were subjected to another ipGTT.

### Quantitative gene expression analysis

Multiple tissues from control and SKM-specific KO mice were harvested and homogenized in TRIzol (ThermoFisher Scientific). Total RNA was isolated using the RNeasy Mini Kit (Qiagen), and RNA quantity was measured with a spectrophotometer (Nanodrop ND 1000; NanoDrop Products, Wilmington, DE). For quantitative real-time PCR (qRT-PCR), 1 μg of total RNA was reversely transcribed (SuperScript III Reverse Transcriptase, Invitrogen) using oligo(dT) primers. qRT-PCR was performed using SYBR Green Master Mix (Applied Biosystems) according to the manufacturer’s instructions. The following primers were used: mouse Barr1: forward, 5’-AAGAAGGCAAGCCCCAAT-3’; reverse 5'-CGCAGGTCAGTGTCACGTAG-3'; mouse Barr2: forward, 5’-AAGTCGAGCCCTAACTGCAA-3’; reverse, 5’-ACAGGGTCCACTTTGTCCAG-3’; mouse β_2_-microglobulin: forward, 5’- GCTATCCAGAAAACCCCTCAAA-3’; reverse, 5’- GGCGGGTGGAACTGTGTTA-3’.

Data were expressed as ΔCt values normalized to the expression of β_2_-microglobulin.

### Western blotting experiments

Tissues were isolated quickly, frozen in liquid nitrogen and stored at -80°C until use. For western blotting studies, frozen tissues were homogenized in ice-cold RIPA buffer (Sigma Aldrich), and protein concentrations were determined using a BCA protein assay (Thermo Fisher Scientific). Protein extracts were separated on 4–12% NuPAGE gels (Thermo Fisher Scientific) and blotted onto nitrocellulose membranes (Bio-Rad). Membranes were blocked for 1 h at room temperature in TBS-T (0.1%) containing 5% BSA. Membranes were then incubated with primary antibodies overnight at 4°C. Following three washing steps in TBS-T (0.1%), membranes were then incubated with HRP-conjugated secondary antibodies for 1 h at room temperature. After washing, proteins were visualized with SuperSignal West Dura Extended Duration Substrate (Thermo Fisher Scientific) on the c600 Imaging System Imager (Azure Biosystems). Immunoreactive bands were quantified using Image J Software (NIH). Uncropped images of the blots are shown in S6 Fig.

The following primary antibodies were used: pAKT (S473) rabbit mAb (#4060; Cell Signaling Technology), pAKT (T308) rabbit mAb (#2965; Cell Signaling Technology), total AKT rabbit mAb (#9272; Cell Signaling Technology), and GAPDH rabbit mAb (HRP-linked, #3683; Cell Signaling Technology), pCREB (#9198; Cell Signaling Technology), tCREB (#9197; Cell Signaling Technology), pGS (#3819; Cell Signaling Technology), tGS (#3886; Cell Signaling Technology). Anti-rabbit IgG (HRP-linked) from Cell Signaling Technology (#7074) was used as the secondary antibody. To detect both Barr1 and Barr2, we used a rabbit polyclonal antibody (F431) [40].

### Hyperinsulinemic-euglycemic clamp

Clamp experiments were performed as described previously [41, 42], with minor modifications. Briefly, 5 days prior to the experiment, the left common carotid artery and the right jugular vein of obese male SKM-Barr2-KO and control mice were catheterized under isoflurane anesthesia. Following a 5-h fast, clamps were performed using unrestrained, conscious mice. The clamp procedure consisted of a 120-min tracer equilibration period (-120 to 0 min), followed by a 120-min clamp period (0 to 120 min). A 1.2-μCi bolus of [3-_3_H]glucose (Perkin Elmer) was given at -120 min, followed by a 0.04 μCi/min infusion for 2 h at a pump rate of 1 μl/min (CMA Microdialysis). The insulin clamp was started at 0 min with an infusion of human insulin (4 mU/kg/min) (Humulin R; Eli Lilly) delivered at a pump rate of 0.4 μl/min. Euglycemia (blood glucose: ∼120–160 mg/dl) was maintained during clamps by measuring blood glucose every 10 min and adjusting as necessary an infusion rate of the mix of 40 μCi [^3^H]-glucose in 600 μl of 45% glucose (hot glucose infusion or HOT GINF). The clamp steady state was achieved within 60–70 min. Blood samples were then collected every 10 min from 80 to 120 min and processed to determine blood glucose-specific activity. Mice also received saline-washed erythrocytes from donors throughout the clamp period (3.5 μl/min) to compensate for the loss of blood during the clamp. To estimate insulin-stimulated glucose fluxes in tissues, 2-deoxy-d-[1-_14_C]glucose (Perkin Elmer) was administered as a bolus (10 μCi) at 120 min. Blood samples were collected 2, 5, 10, 15 and 25 min after the bolus injection. At the end of the clamp, animals were anesthetized with sodium pentobarbital (50 mg/kg i.v.), and tissues were isolated immediately and frozen in liquid nitrogen for analysis.

To determine [3-_3_H]glucose-dependent variables, plasma samples were deproteinized using barium hydroxide and zinc sulfate. Rates of glucose production and disappearance were determined using Steele’s non-steady-state equations [43]. Clamp hepatic endogenous glucose production rate (hGP) was determined by subtracting the glucose infusion rate (GIR) from total glucose turnover (Rd). The glucose uptake by tissues and glycogen synthesis rates were calculated as described previously [44].

### Treadmill exercise capacity test

Exercise capacity of overnight fasted mice was tested using a Columbus Instruments rodent treadmill (Model Eco-6M), set at a 10° incline. Total exercise time, distance, maximum speed and work expended were recorded at the time of exhaustion which was defined as the moment the mouse was unable to continue running without repeatedly falling back onto the shock grid at the back end of the treadmill belt. The testing protocol was as follows: 10 min at 10 m/min, then 12 m/min for 5 min. At 15 min, the belt speed was increased to 15 m/min for 3 min and then increased by 1.8 m/min every 3 min until the mouse became exhausted.

### Metabolic studies after exercise testing

Mice were fasted for 4 h and run on a treadmill for 1 h, using the following protocol: 5 min at 10 m/min, 50 min at 15 m/min, and then 5 min at 18 m/min. At the end of the running protocol, a glucose tolerance test was performed. One week later the experiment was repeated, but mice were euthanized 15 min after the i.p. injection of glucose (2 g/kg). SKM tissues were isolated and snap frozen in liquid nitrogen for measurement of glycogen content and western blotting analysis. Glycogen levels were determined using a glycogen assay kit (Cayman Chemical).

### Statistics

Data are expressed as mean ± SEM for the indicated number of observations. Data were assessed for statistical significance by 2-way-ANOVA (repeated measure) tests, followed by the indicated post hoc tests, or by using a two-tailed unpaired Student’s t-test, as appropriate. A P value of less than 0.05 was considered statistically significant. The specific statistical tests that were used are indicated in the figure legends.

## Supporting information

S1 Fig Analysis of Barr1 and Barr2 mRNA and protein expression levels. (A, B, D**)** Barr1 and Barr2 mRNA levels were quantitated by qRT-PCR. In (A), mRNA was prepared from several SKM tissues and other non-SKM tissues of WT mice. Barr1 and Barr2 transcript levels are expressed as ΔCt values following normalization to the expression of β2-microglobulin. (B, D) Selective knockdown of Barr1 (B) or Barr2 (D) mRNA in SKM tissues of SKM-Barr1-KO and SKM-Barr2-KO mice, respectively. (C, E) Representative Western blots demonstrating the loss of SKM Barr1 (C) or Barr2 (E) protein in SKM of SKM-Barr1-KO and SKM-Barr2-KO mice, respectively. Data are shown as mean ± SEM (3 adult male mice per group). **P<0.01, ***P<0.001 (2-way-ANOVA). Quad, quadriceps muscle; GCM, gastrocnemius muscle; TA, tibialis anterior muscle; Sol, soleus muscle; WAT, white adipose tissue.
(TIF)

S2 Fig Treadmill exercise capacity of SKM-Barr1-KO mice. SKM-Barr1-KO and control mice consuming regular chow that had been fasted overnight were run on a treadmill as described under Methods. (A) Total exercise distance. (B) Running time until exhaustion. (C) Maximum speed. (D) Work expended. (E) Body weight. (F) Blood glucose levels before exercise and at the time of exhaustion. (G) Glucose tolerance test performed after an exercise challenge (see Methods and Fig 4A for details). Mice received an i.p. bolus of 2 g/kg glucose at time ‘0’. Data are presented as mean ± SEM (n = 7 mice per group; adult male littermates).
(TIF)

S3 Fig Treadmill exercise capacity of SKM-Barr2-KO mice. SKM-Barr2-KO and control mice consuming regular chow that had been fasted overnight were run on a treadmill as described under Methods. (A) Total exercise distance. (B) Running time until exhaustion. (C) Maximum speed. (D) Work expended. (E) Body weight. (F) Blood glucose levels before exercise and at the time of exhaustion. Data are shown as mean ± SEM (n = 5 or 6 mice per group; adult male littermates).
(TIF)

S4 Fig Metabolic characterization of inducible SKM-Barr1&2-KO mice. Barr1^fl/fl^ &Barr2^fl/fl^ mice harboring the HSA-Cre(ER^T2^) transgene were injected with tamoxifen, as described under Methods, resulting in the deletion of both Barr1 and Barr2 in SKM (SKM-Barr1&2-iKO mice). Cre-negative littermates served as control animals. (A) Representative Western blot confirming the relative lack of Barr1 and Barr2 protein in SKM- Barr1&2-iKO mice. (B-I) Metabolic analysis of SKM-Barr1&2-iKO mice and control littermates maintained on normal chow (B-E) or a HFD for at least 8 weeks (F-I). (B, F) Body weights. (C, G) Fasting and fed blood glucose levels. (D, H) Glucose tolerance tests. (E, I) Insulin tolerance tests (0.75 IU/kg i.p.). Initial blood glucose levels were set to 100% (actual basal blood glucose levels were (in mg/dl): 129 ± 7 vs. 139 ± 5 (E) and 160 ± 6 vs.177 ± 6 (I) for control vs. SKM-Barr1&2-iKO mice, respectively). Data are shown as mean ± SEM (n = 10–12 mice per group; adult male littermates). iKO, inducible KO. Two-way-ANOVA repeated measure tests showed no significant differences between control and SKM-Barr1&2-iKO mice in any of the metabolic tests.
(TIF)

S5 Fig Metabolic characterization of constitutive SKM-Barr1&2-KO mice. Barr1^fl/fl^&Barr2^fl/fl^ mice carrying the HSA-Cre transgene (SKM-Barr1&2-cKO mice) and their Cre-negative control littermates were subjected to a series of *in vivo* metabolic tests. (A-H) Metabolic analysis of SKM-Barr1&2-cKO mice and control littermates maintained on normal chow (A-D) or a HFD for at least 8 weeks (E-H). (A, E) Body weights. (B, F) Fasting and fed blood glucose levels. (C, G) Glucose tolerance tests. (D, H) Insulin tolerance tests (0.75 IU/kg i.p.). Initial blood glucose levels were set to 100% (actual basal blood glucose levels were (in mg/dl): 157 ± 6 vs. 154 ± 9 (D) and 212 ± 17 vs. 205 ± 18 (H) for control vs. SKM-Barr1&2-cKO mice, respectively). Data are presented as mean ± SEM (n = 6–9 mice per group; adult male littermates). cKO, constitutive KO. Two-way-ANOVA repeated measure tests showed no significant differences between control and SKM-Barr1&2-cKO mice in any of the metabolic tests.
(TIF)

S6 Fig Uncropped western blot images and Barr1/2 antibody ‘calibration curves’. (A-D) Original blots for Fig 2K (A), Fig 4C (B), Fig 4D (C) and S1C, S1E and S4A Figs (D). A rabbit polyclonal antibody (F431) was used to detect both Barr1 and Barr2. (E) Barr1/2 Western blot from S6D Fig was repeated including defined amounts of purified Barr1 and Barr2 proteins as loading controls. Protein concentrations of quadriceps muscle samples were determined using a BCA protein assay, and 7.5 μg of protein were loaded per lane. (F) Barr1 and Barr2 calibration curves were generated by loading increasing amounts of purified Barr1 and Barr2 proteins. The blot clearly shows that the F431 Barr1/2 antibody recognizes Barr2 with significantly greater sensitivity than Barr1.
(TIF)

## References

[1] Cho NH, Shaw JE, Karuranga S, Huang Y, da Rocha Fernandes JD, Ohlrogge AW, et al IDF Diabetes Atlas: Global estimates of diabetes prevalence for 2017 and projections for 2045. Diabetes Res Clin Pract. 2018;138:271–81. Epub 2018/03/03. 10.1016/j.diabres.2018.02.023 . 29496507

[2] DeFronzo RA, Tripathy D. Skeletal muscle insulin resistance is the primary defect in type 2 diabetes. Diabetes care. 2009;32 Suppl 2:S157–63. 10.2337/dc09-S302 19875544 PMC2811436

[3] Gonzalez-Muniesa P, Martinez-Gonzalez MA, Hu FB, Despres JP, Matsuzawa Y, Loos RJF, et al Obesity. Nature reviews Disease primers. 2017;3:17034 Epub 2017/06/16. 10.1038/nrdp.2017.34 . 28617414

[4] Kusminski CM, Bickel PE, Scherer PE. Targeting adipose tissue in the treatment of obesity-associated diabetes. Nat Rev Drug Discov. 2016;15(9):639–60. Epub 2016/06/04. 10.1038/nrd.2016.75 . 27256476

[5] Thiebaud D, Jacot E, DeFronzo RA, Maeder E, Jequier E, Felber JP. The effect of graded doses of insulin on total glucose uptake, glucose oxidation, and glucose storage in man. Diabetes. 1982;31(11):957–63. Epub 1982/11/01. 10.2337/diacare.31.11.957 . 6757014

[6] Coureuil M, Lecuyer H, Scott MG, Boularan C, Enslen H, Soyer M, et al Meningococcus Hijacks a beta2-adrenoceptor/beta-Arrestin pathway to cross brain microvasculature endothelium. Cell. 2010;143(7):1149–60. Epub 2010/12/25. 10.1016/j.cell.2010.11.035 . 21183077

[7] Luan B, Zhao J, Wu H, Duan B, Shu G, Wang X, et al Deficiency of a beta-arrestin-2 signal complex contributes to insulin resistance. Nature. 2009;457(7233):1146–9. 10.1038/nature07617 . 19122674

[8] Zhuang LN, Hu WX, Zhang ML, Xin SM, Jia WP, Zhao J, et al Beta-arrestin-1 protein represses diet-induced obesity. J Biol Chem. 2011;286(32):28396–402. 10.1074/jbc.M111.223206 21543334 PMC3151082

[9] Wang Y, Jin L, Song Y, Zhang M, Shan D, Liu Y, et al beta-arrestin 2 mediates cardiac ischemia-reperfusion injury via inhibiting GPCR-independent cell survival signalling. Cardiovasc Res. 2017;113(13):1615–26. Epub 2017/10/11. 10.1093/cvr/cvx147 . 29016703

[10] Zhu L, Rossi M, Cui Y, Lee RJ, Sakamoto W, Perry NA, et al Hepatic beta-arrestin 2 is essential for maintaining euglycemia. J Clin Invest. 2017;127(8):2941–5. Epub 2017/06/27. 10.1172/JCI92913 28650340 PMC5531395

[11] Zhu L, Almaca J, Dadi PK, Hong H, Sakamoto W, Rossi M, et al beta-arrestin-2 is an essential regulator of pancreatic beta-cell function under physiological and pathophysiological conditions. Nat Commun. 2017;8:14295 Epub 2017/02/02. 10.1038/ncomms14295 28145434 PMC5296650

[12] Ravier MA, Leduc M, Richard J, Linck N, Varrault A, Pirot N, et al beta-Arrestin2 plays a key role in the modulation of the pancreatic beta cell mass in mice. Diabetologia. 2014;57(3):532–41. Epub 2013/12/10. 10.1007/s00125-013-3130-7 . 24317793

[13] Pierce KL, Premont RT, Lefkowitz RJ. Seven-transmembrane receptors. Nat Rev Mol Cell Biol. 2002;3(9):639–50. Epub 2002/09/05. 10.1038/nrm908 . 12209124

[14] Srivastava A, Gupta B, Gupta C, Shukla AK. Emerging Functional Divergence of beta-Arrestin Isoforms in GPCR Function. Trends Endocrinol Metab. 2015;26(11):628–42. Epub 2015/10/17. 10.1016/j.tem.2015.09.001 . 26471844

[15] Lee AD, Hansen PA, Schluter J, Gulve EA, Gao J, Holloszy JO. Effects of epinephrine on insulin-stimulated glucose uptake and GLUT-4 phosphorylation in muscle. Am J Physiol. 1997;273(3 Pt 1):C1082–7. Epub 1997/10/08. 10.1152/ajpcell.1997.273.3.C1082 . 9316430

[16] Opie LH. Effect of beta-adrenergic blockade on biochemical and metabolic response to exercise. Am J Cardiol. 1985;55(10):95D–100D. Epub 1985/04/26. 10.1016/0002-9149(85)91062-8 . 2859797

[17] Schmid CL, Bohn LM. Physiological and pharmacological implications of beta-arrestin regulation. Pharmacol Ther. 2009;121(3):285–93. Epub 2008/12/23. 10.1016/j.pharmthera.2008.11.005 19100766 PMC2656564

[18] Luttrell LM, Gesty-Palmer D. Beyond desensitization: physiological relevance of arrestin-dependent signaling. Pharmacol Rev. 2010;62(2):305–30. Epub 2010/04/30. 10.1124/pr.109.002436 20427692 PMC2879915

[19] Whalen EJ, Rajagopal S, Lefkowitz RJ. Therapeutic potential of beta-arrestin- and G protein-biased agonists. Trends Mol Med. 2011;17(3):126–39. Epub 2010/12/25. 10.1016/j.molmed.2010.11.004 21183406 PMC3628754

[20] Kenakin T, Christopoulos A. Signalling bias in new drug discovery: detection, quantification and therapeutic impact. Nat Rev Drug Discov. 2013;12(3):205–16. Epub 2013/02/16. 10.1038/nrd3954 . 23411724

[21] Zhuang LN, Hu WX, Xin SM, Zhao J, Pei G. Beta-arrestin-1 protein represses adipogenesis and inflammatory responses through its interaction with peroxisome proliferator-activated receptor-gamma (PPARgamma). J Biol Chem. 2011;286(32):28403–13. Epub 2011/06/28. 10.1074/jbc.M111.256099 21700709 PMC3151083

[22] Pydi SP, Jain S, Tung W, Cui Y, Zhu L, Sakamoto W, et al Adipocyte beta-arrestin-2 is essential for maintaining whole body glucose and energy homeostasis. Nat Commun. 2019;10(1):2936 Epub 2019/07/05. 10.1038/s41467-019-11003-4 31270323 PMC6610117

[23] Barella LF, Rossi M, Zhu L, Cui Y, Mei FC, Cheng X, et al beta-Cell-intrinsic beta-arrestin 1 signaling enhances sulfonylurea-induced insulin secretion. J Clin Invest. 2019;130 Epub 2019/06/12. 10.1172/JCI126309 . 31184597 PMC6715363

[24] Kim J, Grotegut CA, Wisler JW, Li T, Mao L, Chen M, et al beta-arrestin 1 regulates beta2-adrenergic receptor-mediated skeletal muscle hypertrophy and contractility. Skeletal muscle. 2018;8(1):39 Epub 2018/12/29. 10.1186/s13395-018-0184-8 30591079 PMC6309084

[25] Schuler M, Ali F, Metzger E, Chambon P, Metzger D. Temporally controlled targeted somatic mutagenesis in skeletal muscles of the mouse. Genesis (New York, NY: 2000). 2005;41(4):165–70. Epub 2005/03/25. 10.1002/gene.20107 . 15789425

[26] Pierce KL, Lefkowitz RJ. Classical and new roles of beta-arrestins in the regulation of G-protein-coupled receptors. Nat Rev Neurosci. 2001;2(10):727–33. Epub 2001/10/05. 10.1038/35094577 . 11584310

[27] Regard JB, Sato IT, Coughlin SR. Anatomical profiling of G protein-coupled receptor expression. Cell. 2008;135(3):561–71. Epub 2008/11/06. 10.1016/j.cell.2008.08.040 18984166 PMC2590943

[28] Bruno NE, Kelly KA, Hawkins R, Bramah-Lawani M, Amelio AL, Nwachukwu JC, et al Creb coactivators direct anabolic responses and enhance performance of skeletal muscle. EMBO J. 2014;33(9):1027–43. Epub 2014/03/29. 10.1002/embj.201386145 24674967 PMC4193935

[29] Katz A, Broberg S, Sahlin K, Wahren J. Leg glucose uptake during maximal dynamic exercise in humans. Am J Physiol. 1986;251(1 Pt 1):E65–70. Epub 1986/07/01. 10.1152/ajpendo.1986.251.1.E65 . 3728665

[30] Sylow L, Kleinert M, Richter EA, Jensen TE. Exercise-stimulated glucose uptake—regulation and implications for glycaemic control. Nat Rev Endocrinol. 2017;13(3):133–48. Epub 2016/11/04. 10.1038/nrendo.2016.162 . 27739515

[31] Sato M, Dehvari N, Oberg AI, Dallner OS, Sandstrom AL, Olsen JM, et al Improving type 2 diabetes through a distinct adrenergic signaling pathway involving mTORC2 that mediates glucose uptake in skeletal muscle. Diabetes. 2014;63(12):4115–29. Epub 2014/07/11. 10.2337/db13-1860 . 25008179

[32] Nevzorova J, Evans BA, Bengtsson T, Summers RJ. Multiple signalling pathways involved in beta2-adrenoceptor-mediated glucose uptake in rat skeletal muscle cells. Br J Pharmacol. 2006;147(4):446–54. Epub 2006/01/18. 10.1038/sj.bjp.0706626 16415914 PMC1616992

[33] Elayan H, Milic M, Sun P, Gharaibeh M, Ziegler MG. Chronic beta2 adrenergic agonist, but not exercise, improves glucose handling in older type 2 diabetic mice. Cell Mol Neurobiol. 2012;32(5):871–7. Epub 2012/03/17. 10.1007/s10571-012-9819-1 . 22422105 PMC11498512

[34] Ngala RA, O'Dowd J, Wang SJ, Stocker C, Cawthorne MA, Arch JR. Beta2-adrenoceptors and non-beta-adrenoceptors mediate effects of BRL37344 and clenbuterol on glucose uptake in soleus muscle: studies using knockout mice. Br J Pharmacol. 2009;158(7):1676–82. Epub 2009/11/17. 10.1111/j.1476-5381.2009.00472.x 19912225 PMC2801208

[35] Castle A, Yaspelkis BB 3rd, Kuo CH, Ivy JL. Attenuation of insulin resistance by chronic beta2-adrenergic agonist treatment possible muscle specific contributions. Life Sci. 2001;69(5):599–611. Epub 2001/08/21. 10.1016/s0024-3205(01)01149-3 . 11510954

[36] Bowe JE, Franklin ZJ, Hauge-Evans AC, King AJ, Persaud SJ, Jones PM. Metabolic phenotyping guidelines: assessing glucose homeostasis in rodent models. J Endocrinol. 2014;222(3):G13–25. Epub 2014/07/25. 10.1530/JOE-14-0182 . 25056117

[37] Ayala JE, Bracy DP, McGuinness OP, Wasserman DH. Considerations in the design of hyperinsulinemic-euglycemic clamps in the conscious mouse. Diabetes. 2006;55(2):390–7. Epub 2006/01/31. 10.2337/diabetes.55.02.06.db05-0686 . 16443772

[38] Wu M, Falasca M, Blough ER. Akt/protein kinase B in skeletal muscle physiology and pathology. Journal of cellular physiology. 2011;226(1):29–36. Epub 2010/07/31. 10.1002/jcp.22353 . 20672327

[39] Urs NM, Gee SM, Pack TF, McCorvy JD, Evron T, Snyder JC, et al Distinct cortical and striatal actions of a beta-arrestin-biased dopamine D2 receptor ligand reveal unique antipsychotic-like properties. Proc Natl Acad Sci U S A. 2016;113(50):E8178–e86. Epub 2016/12/03. 10.1073/pnas.1614347113 27911814 PMC5167191

[40] Perry NA, Kaoud TS, Ortega OO, Kaya AI, Marcus DJ, Pleinis JM, et al Arrestin-3 scaffolding of the JNK3 cascade suggests a mechanism for signal amplification. Proc Natl Acad Sci U S A. 2019;116(3):810–5. Epub 2018/12/29. 10.1073/pnas.1819230116 30591558 PMC6338856

[41] Ayala JE, Bracy DP, Malabanan C, James FD, Ansari T, Fueger PT, et al Hyperinsulinemic-euglycemic clamps in conscious, unrestrained mice. J Vis Exp. 2011;(57). Epub 2011/12/01. 10.3791/3188 22126863 PMC3308587

[42] Godlewski G, Jourdan T, Szanda G, Tam J, Cinar R, Harvey-White J, et al Mice lacking GPR3 receptors display late-onset obese phenotype due to impaired thermogenic function in brown adipose tissue. Sci Rep. 2015;5:14953 Epub 2015/10/13. 10.1038/srep14953 26455425 PMC4601089

[43] Steele R, Wall JS, De Bodo RC, Altszuler N. Measurement of size and turnover rate of body glucose pool by the isotope dilution method. Am J Physiol. 1956;187(1):15–24. Epub 1956/10/01. 10.1152/ajplegacy.1956.187.1.15 . 13362583

[44] Youn JH, Kim JK, Buchanan TA. Time courses of changes in hepatic and skeletal muscle insulin action and GLUT4 protein in skeletal muscle after STZ injection. Diabetes. 1994;43(4):564–71. Epub 1994/04/01. 10.2337/diab.43.4.564 . 8138062

