# Supplementary figures and images for: Metabolic effects of skeletal muscle-specific deletion of beta-arrestin-1 and -2 in mice
Source: PLoS Genet. 2019 Oct 17;15(10):e1008424. doi: 10.1371/journal.pgen.1008424 (PMC6818801; doi:10.1371/journal.pgen.1008424)

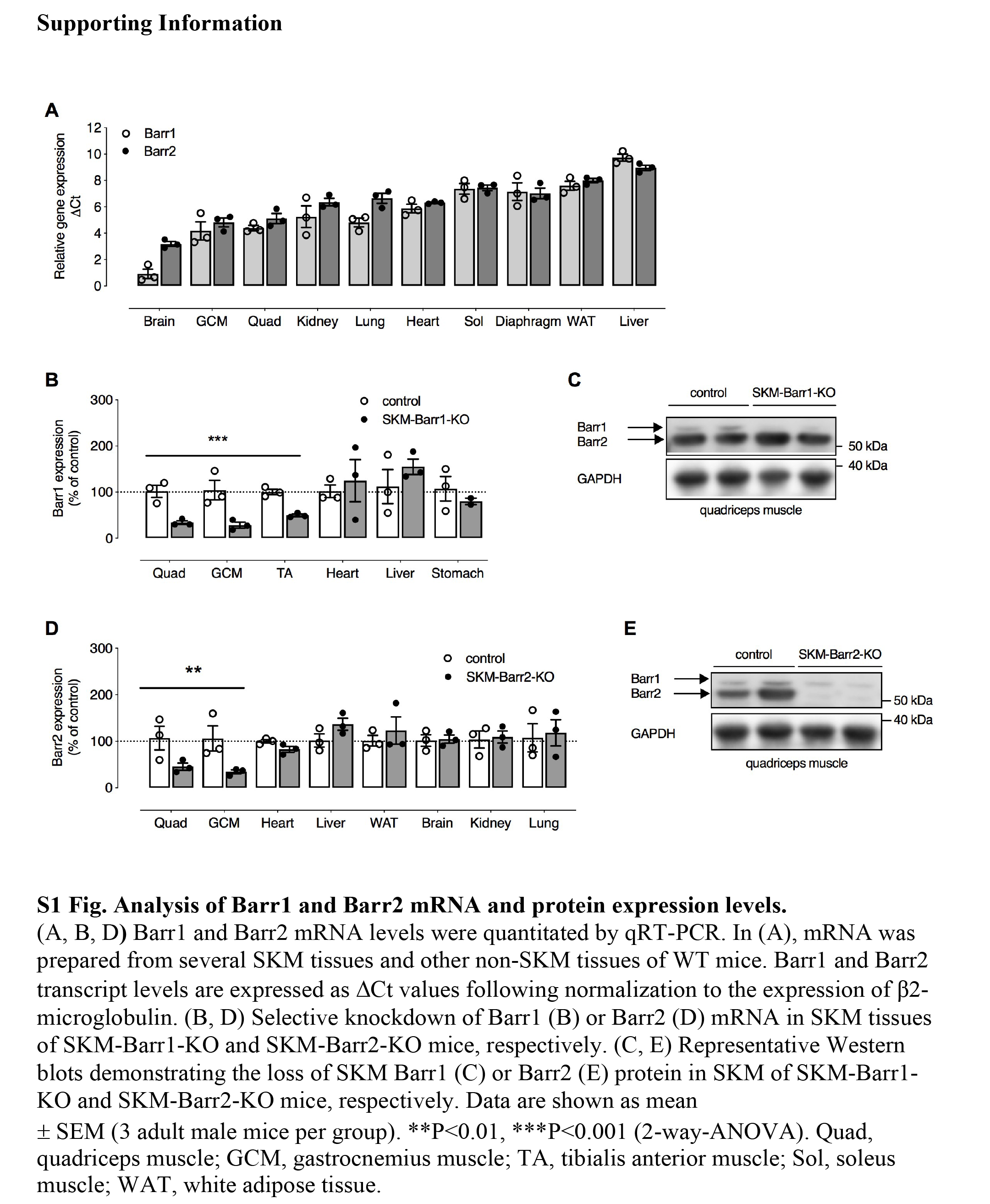

Supplement: S1 Fig — (A, B, D) Barr1 and Barr2 mRNA levels were quantitated by qRT-PCR. In (A), mRNA was prepared from several SKM tissues and other non-SKM tissues of WT mice. Barr1 and Barr2 transcript levels are expressed as ΔCt values following normalization to the expression of β2-microglobulin. (B, D) Selective knockdown of Barr1 (B) or Barr2 (D) mRNA in SKM tissues of SKM-Barr1-KO and SKM-Barr2-KO mice, respectively. (C, E) Representative Western blots demonstrating the loss of SKM Barr1 (C) or Barr2 (E) protein in SKM of SKM-Barr1-KO and SKM-Barr2-KO mice, respectively. Data are shown as mean ± SEM (3 adult male mice per group). **P<0.01, ***P<0.001 (2-way-ANOVA). Quad, quadriceps muscle; GCM, gastrocnemius muscle; TA, tibialis anterior muscle; Sol, soleus muscle; WAT, white adipose tissue. (TIF) [file pgen.1008424.s001.tif]

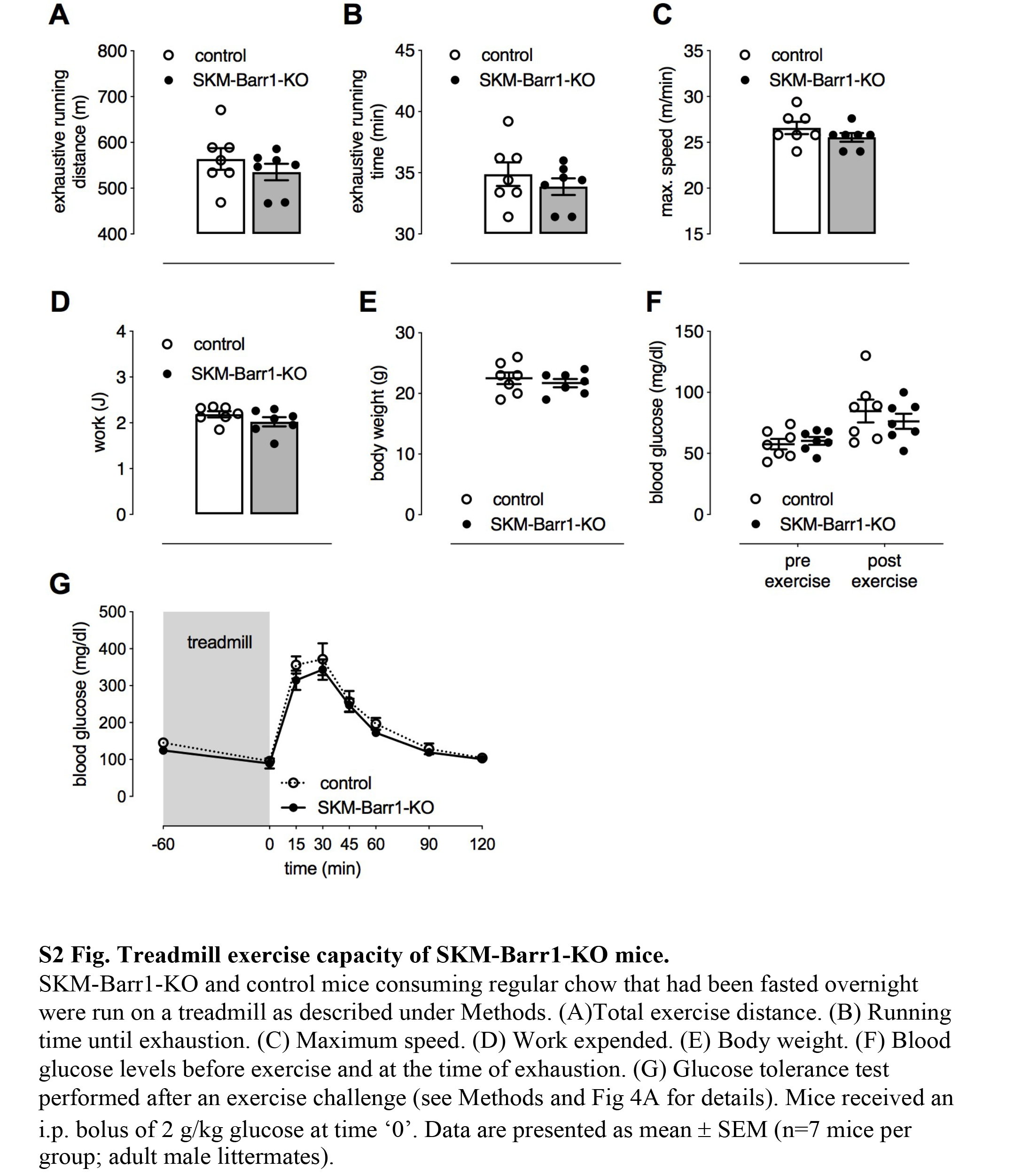

Supplement: S2 Fig — SKM-Barr1-KO and control mice consuming regular chow that had been fasted overnight were run on a treadmill as described under Methods. (A) Total exercise distance. (B) Running time until exhaustion. (C) Maximum speed. (D) Work expended. (E) Body weight. (F) Blood glucose levels before exercise and at the time of exhaustion. (G) Glucose tolerance test performed after an exercise challenge (see Methods and Fig 4A for details). Mice received an i.p. bolus of 2 g/kg glucose at time ‘0’. Data are presented as mean ± SEM (n = 7 mice per group; adult male littermates). (TIF) [file pgen.1008424.s002.tif]

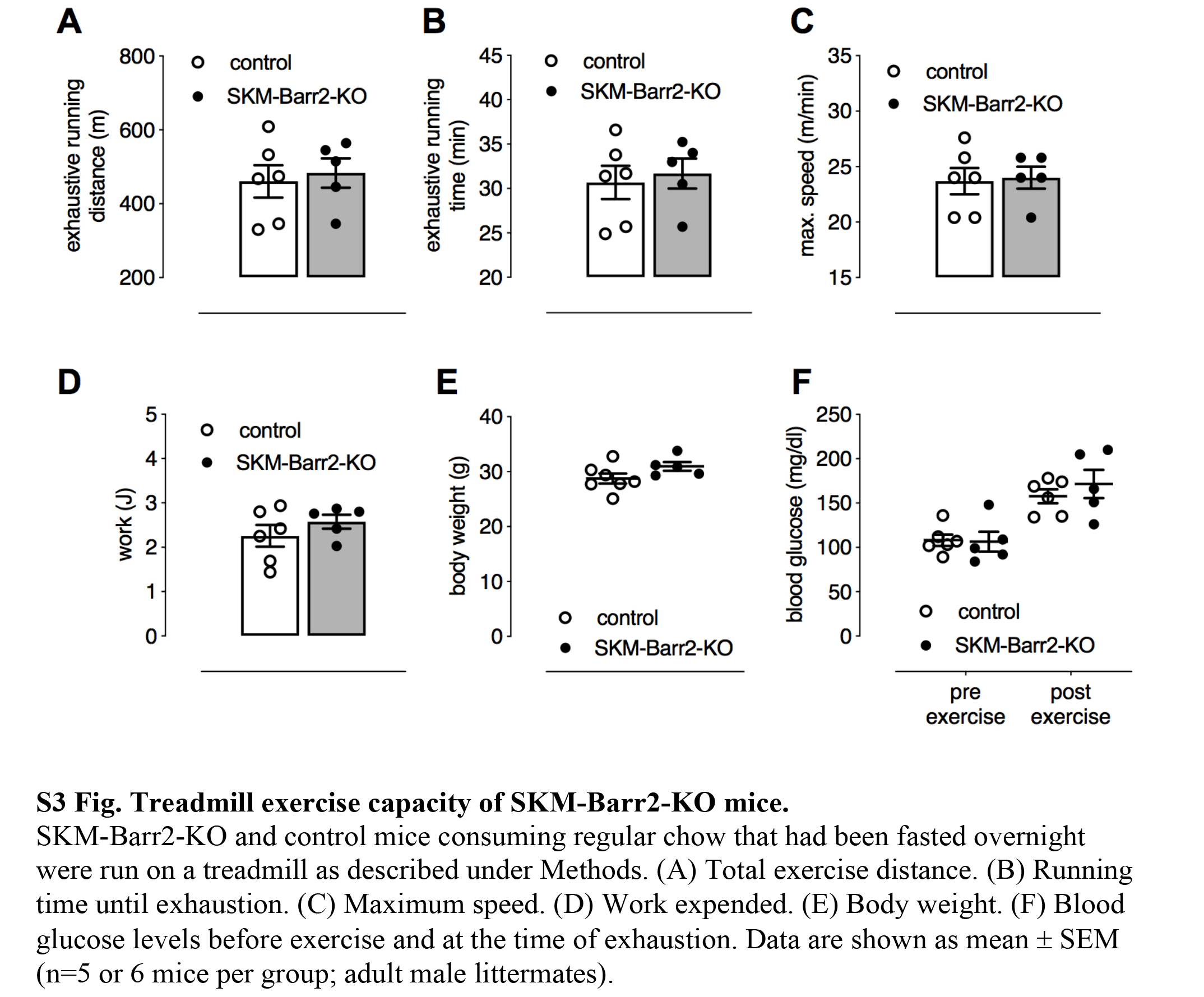

Supplement: S3 Fig — SKM-Barr2-KO and control mice consuming regular chow that had been fasted overnight were run on a treadmill as described under Methods. (A) Total exercise distance. (B) Running time until exhaustion. (C) Maximum speed. (D) Work expended. (E) Body weight. (F) Blood glucose levels before exercise and at the time of exhaustion. Data are shown as mean ± SEM (n = 5 or 6 mice per group; adult male littermates). (TIF) [file pgen.1008424.s003.tif]

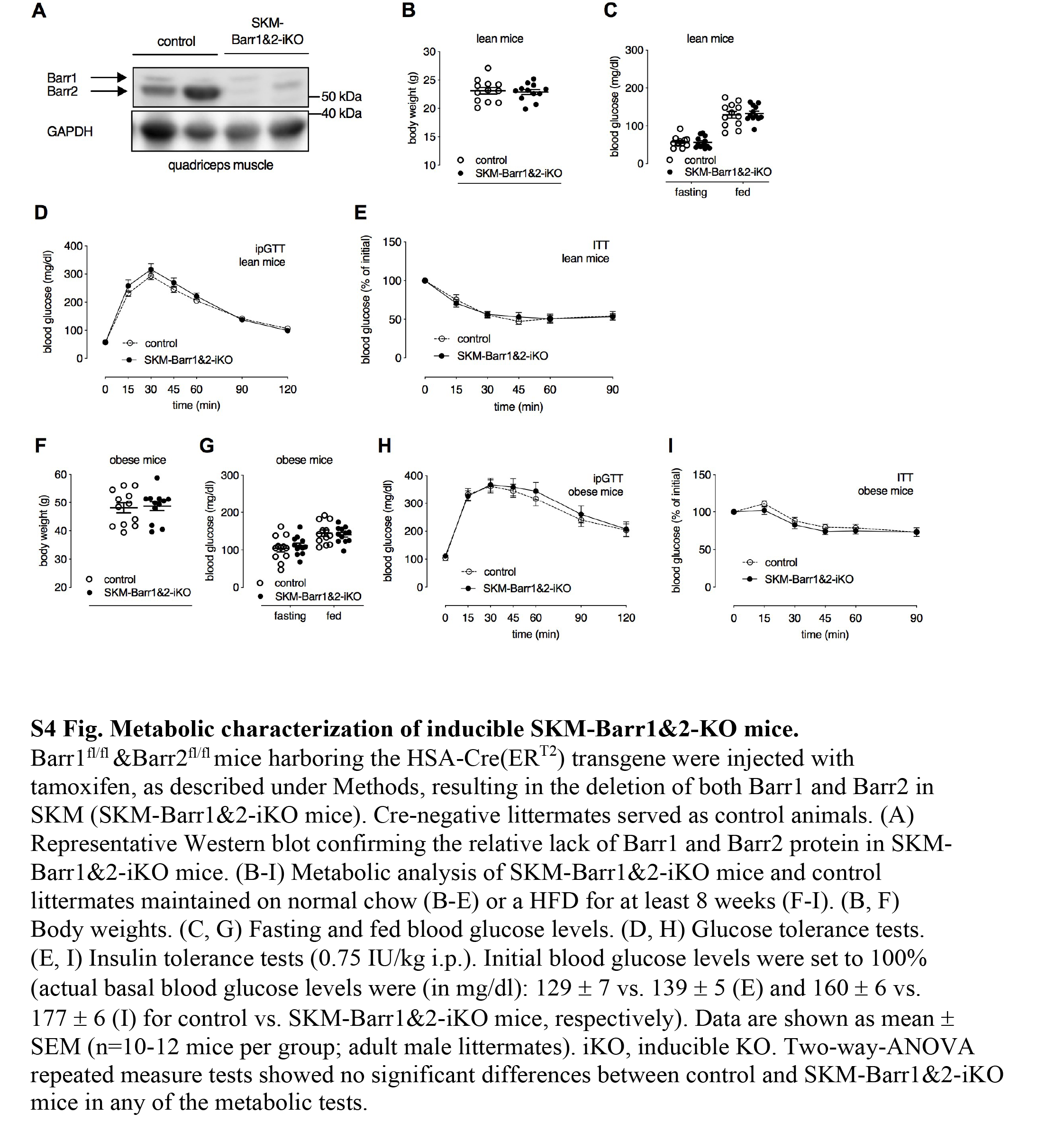

Supplement: S4 Fig — Barr1fl/fl &Barr2fl/fl mice harboring the HSA-Cre(ERT2) transgene were injected with tamoxifen, as described under Methods, resulting in the deletion of both Barr1 and Barr2 in SKM (SKM-Barr1&2-iKO mice). Cre-negative littermates served as control animals. (A) Representative Western blot confirming the relative lack of Barr1 and Barr2 protein in SKM- Barr1&2-iKO mice. (B-I) Metabolic analysis of SKM-Barr1&2-iKO mice and control littermates maintained on normal chow (B-E) or a HFD for at least 8 weeks (F-I). (B, F) Body weights. (C, G) Fasting and fed blood glucose levels. (D, H) Glucose tolerance tests. (E, I) Insulin tolerance tests (0.75 IU/kg i.p.). Initial blood glucose levels were set to 100% (actual basal blood glucose levels were (in mg/dl): 129 ± 7 vs. 139 ± 5 (E) and 160 ± 6 vs.177 ± 6 (I) for control vs. SKM-Barr1&2-iKO mice, respectively). Data are shown as mean ± SEM (n = 10–12 mice per group; adult male littermates). iKO, inducible KO. Two-way-ANOVA repeated measure tests showed no significant differences between control and SKM-Barr1&2-iKO mice in any of the metabolic tests. (TIF) [file pgen.1008424.s004.tif]

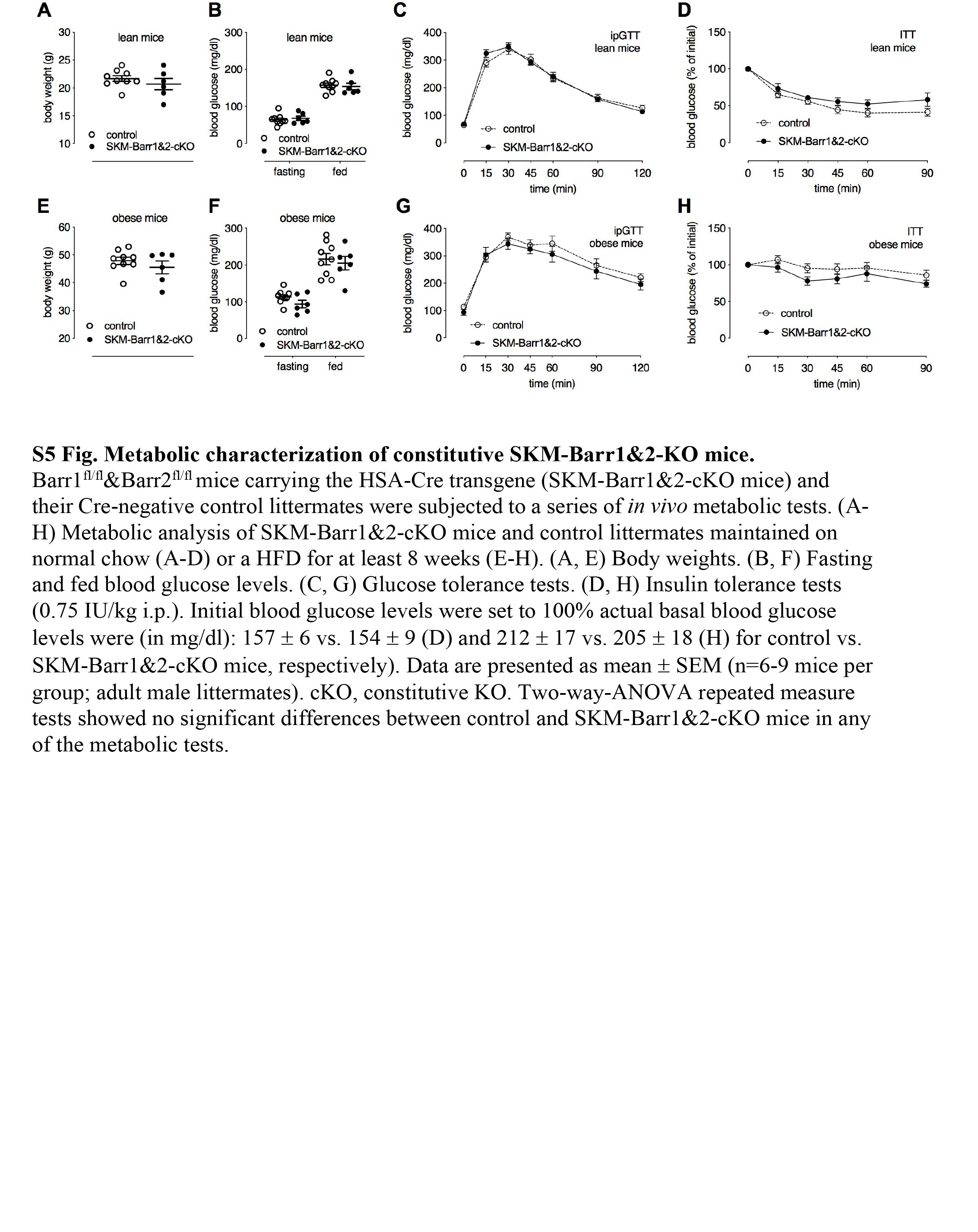

Supplement: S5 Fig — Barr1fl/fl&Barr2fl/fl mice carrying the HSA-Cre transgene (SKM-Barr1&2-cKO mice) and their Cre-negative control littermates were subjected to a series of in vivo metabolic tests. (A-H) Metabolic analysis of SKM-Barr1&2-cKO mice and control littermates maintained on normal chow (A-D) or a HFD for at least 8 weeks (E-H). (A, E) Body weights. (B, F) Fasting and fed blood glucose levels. (C, G) Glucose tolerance tests. (D, H) Insulin tolerance tests (0.75 IU/kg i.p.). Initial blood glucose levels were set to 100% (actual basal blood glucose levels were (in mg/dl): 157 ± 6 vs. 154 ± 9 (D) and 212 ± 17 vs. 205 ± 18 (H) for control vs. SKM-Barr1&2-cKO mice, respectively). Data are presented as mean ± SEM (n = 6–9 mice per group; adult male littermates). cKO, constitutive KO. Two-way-ANOVA repeated measure tests showed no significant differences between control and SKM-Barr1&2-cKO mice in any of the metabolic tests. (TIF) [file pgen.1008424.s005.tif]

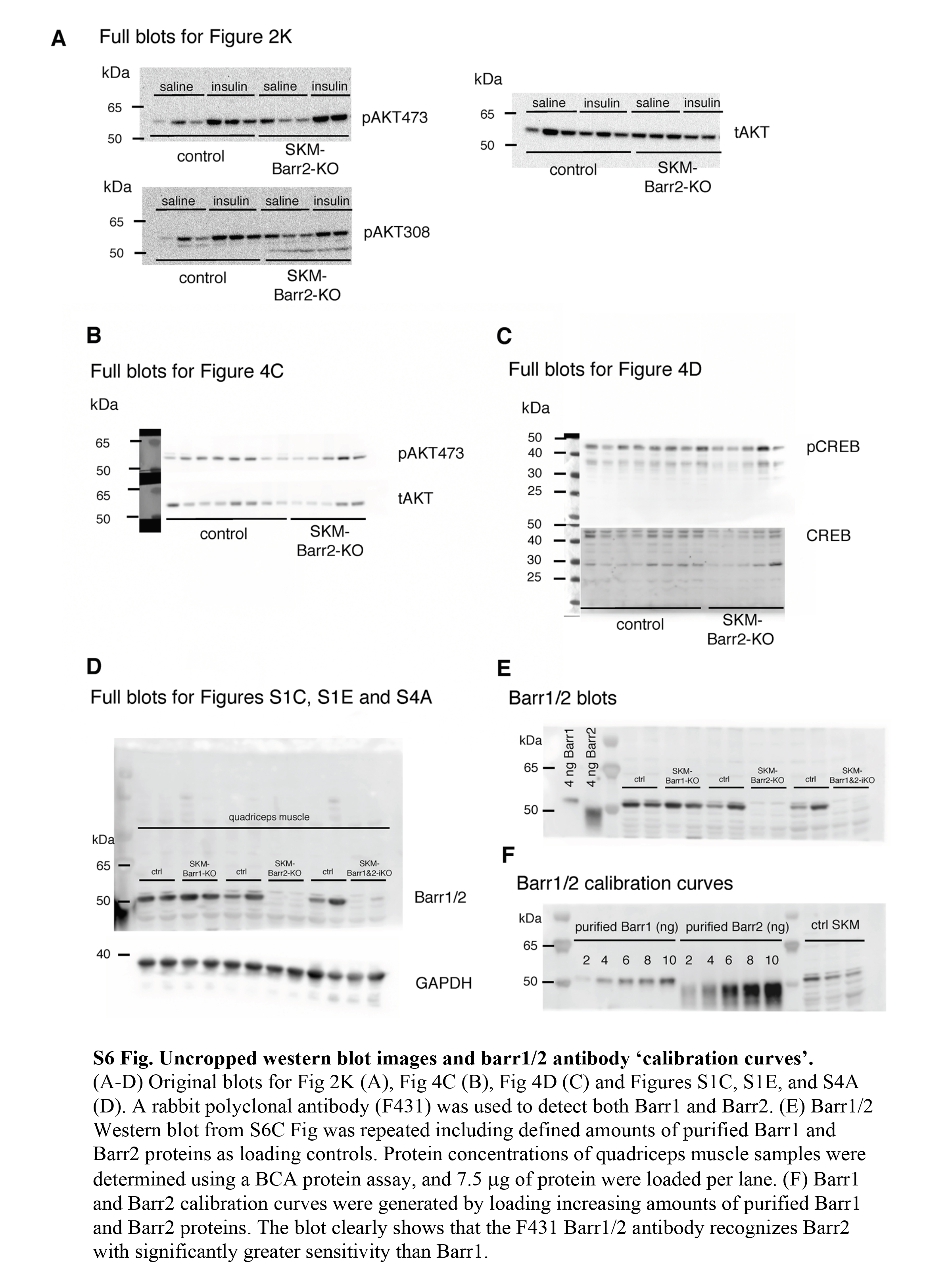

Supplement: S6 Fig — (A-D) Original blots for Fig 2K (A), Fig 4C (B), Fig 4D (C) and S1C, S1E and S4A Figs (D). A rabbit polyclonal antibody (F431) was used to detect both Barr1 and Barr2. (E) Barr1/2 Western blot from S6D Fig was repeated including defined amounts of purified Barr1 and Barr2 proteins as loading controls. Protein concentrations of quadriceps muscle samples were determined using a BCA protein assay, and 7.5 μg of protein were loaded per lane. (F) Barr1 and Barr2 calibration curves were generated by loading increasing amounts of purified Barr1 and Barr2 proteins. The blot clearly shows that the F431 Barr1/2 antibody recognizes Barr2 with significantly greater sensitivity than Barr1. (TIF) [file pgen.1008424.s006.tif]
